# The Health Care Trajectories of Older People in Foster Families: Protocol for an Observational Study

**DOI:** 10.2196/40604

**Published:** 2023-02-08

**Authors:** Denis Boucaud-Maitre, Roxane Villeneuve, Nadine Simo-Tabué, Jean-François Dartigues, Helene Amieva, Maturin Tabué-Teguo

**Affiliations:** 1 Geriatric Units Centre Hospitalo-Universitaire de Guadeloupe Pointe-à-Pitre Guadeloupe; 2 Inserm U1219 Bordeaux Population Health Center University of Bordeaux Bordeaux France

**Keywords:** older people, foster family, hospitalization, geriatric syndromes, mortality, quality of life

## Abstract

**Background:**

With aging of the population, the search for alternative models of care adapted to older people with dependency is necessary. In this setting, foster families (1-3 older people per family) could be an alternative to nursing homes, residential care facilities, or community- and home-based care.

**Objective:**

The KArukera Study of Ageing in Foster Families is a prospective cohort study designed to investigate the care pathways of older people with dependency in foster care over a year. The 1-year hospitalization rate (main objective), cost of hospitalization, incidence of mortality, prevalence of geriatric syndromes, and quality of life of residents will be assessed. Quality of life and burnout of their respective foster caregivers will also be studied.

**Methods:**

This study cohort will include 250 older people living in foster families in Guadeloupe (French West Indies), as well as their respective foster caregivers. Both older people and caregivers will be interviewed concurrently on site at three time points: (1) at baseline, (2) at 6 months, and (3) at 12 months. For older people, we will collect anthropometric measures, cognitive impairment, depressive and anxiety symptoms, functional abilities, physical frailty, information on general health status, quality of life, and care pathways (hospitalization, mortality, and medical and paramedical consultations). We will also assess the quality of life and burnout symptoms of family caregivers at each follow-up. A phone update of vital status (alive or death) and care pathways of residents will be carried out at 3 and 9 months after the baseline examination.

**Results:**

Recruitment opened in September 2020 and ended in May 2021, with 109 older people recruited and 56 respective foster caregivers. The 1-year follow-up was ended in June 2022. Data analyses are ongoing and the first results are expected to be published in May 2023.

**Conclusions:**

Foster families are a potentially innovative way to accommodate dependent older people. This study could help define the clinical profile of older people adapted to foster families in the transition from frailty to dependency. The effectiveness of foster families, in terms of hospitalizations and mortality, will be compared with other models of care, particularly nursing homes. In this setting, a twin study carried out in nursing homes in Guadeloupe with similar aims and outcomes will be conducted. Beyond mortality and morbidity, the numerous outcomes will allow us to assess the evolution of geriatric syndromes over time.

**Trial Registration:**

ClinicalTrials.gov NCT04545775; https://clinicaltrials.gov/ct2/show/NCT04545775

**International Registered Report Identifier (IRRID):**

DERR1-10.2196/40604

## Introduction

The Caribbean region is a heterogeneous canvas of autonomous states and dependent territories of sovereign states. In this area, the French West Indies constitute a specific cultural topic. A total of 90% of its population is of African descent. Although the official language is French, the medical and cultural realities are quite different from mainland France. There is little epidemiological data on this population, despite strong specificities, in terms of prevalence of specific diseases (hypertension, diabetes, etc), specific genetic risk factors, and socioeconomic characteristics or access to health care [1].

On January 1, 2016, there were officially 394,110 inhabitants in Guadeloupe, a number that has been decreasing by 0.5% every year since 2011 [2]. This decrease is the result of both a slowdown in demographic growth and a constant negative net migration rate, due to high unemployment rates and lack of economic prospects. As a result, by 2030, people older than 65 could represent 28% of the population [2] and the number of dependent older people should double. This older population will have age-related medical problems, especially chronic (hypertension, diabetes, and cancer) or neuropsychiatric pathologies. An increase in dependency and its associated consequences, such as an increase in hospitalizations and nursing home admissions, is to be expected. Early detection is lacking, and general practitioners cannot compensate for the shortage of medical specialists. In the current context, the economic, medical, social, and human stakes are considerable and finding the appropriate care option for older people with dependency has become a critical issue.

Despite strong family support, living at home is sometimes difficult or impossible for seniors and their caregivers [3] (heavy dependency, exhaustion of caregivers, regular decompensation of chronic pathologies, etc). Moreover, social isolation, social vulnerability, and loneliness among older people living at home [4,5] increase the risk of adverse health events, notably mortality [6]. The most developed and successful model for dependent persons in high- and middle-income countries is the nursing homes. However, the transition from one’s home to a nursing home usually disrupts residents’ social life and lifestyle, and institutionalized older people experience a steeper cognitive decline than community-dwelling people [7], as well as a drop in quality of life [8] and an increase in psychotropic drug use [9]. For these reasons, we need to study alternative and transitional care models between home care and nursing home care, such as foster families. Because fostered older people (while still moving out of their own homes) are taken care of within a family context instead of a nursing home or other collective settings, foster care placement processes could be an alternative to institutionalization. This model has developed to a greater extent over the last few years in Guadeloupe. Nearly 200 foster families care for about 300 dependent older people, whereas there are 21 nursing homes for approximately 1500 residents. Foster caregivers, after getting official approval and attending a 20-hour training course, usually accommodate 1 to 3 people in exchange for a monthly fee. Their mission is to ensure the well-being of their residents and to enable them to maintain close social and family relationships. Other professionals may also provide additional nursing care if necessary. Little is known about the characteristics of foster caregivers and their residents. Two decades ago, a study from Cébula and Horel [10] showed that 61% of fostered older people were older than 80 years in France, and two-thirds were women. Every 6 of 10 older people lived at home or with their own family before entering foster care, and foster care was requested by the older person’s household in 63% of cases. Most foster caregivers were women (96%). Two-thirds of the foster caregivers were between 40 and 60 years old, and 16% of them were older than 60 years. Every 8 of 10 foster caregivers were “partnered people,” the majority of them being single-family home owners (95%). Nearly half of the foster caregivers were in a nonemployment situation (inactive, unemployed, integration, and training) before obtaining their foster caregiver license. The cost of foster family accommodation for seniors depends on the number of caregiving hours, because of the length and frequency of stay (in 94% of cases, it is a full time) and geographical location. The monthly fee includes rent, services rendered, a maintenance allowance, and a daily allowance. Monthly cost ranges from €1400 to €2100 (US $1521.98 to US $2282.97) per month [11].

Foster families have also been tried out in others countries (eg, United States, Belgium, Italy, Russia, and Finland), but few scientific studies have assessed its effectiveness and efficiency. A Cochrane meta-analysis published in 2015 compiled 10 studies comparing home care or foster care with nursing home care for 16,377 older participants [12]. Because of the methodological limitations of the studies included, the meta-analysis failed to determine whether or not this type of accommodation was the most efficient in terms of mortality reduction, physical function, quality of life, or hospitalization rates. Indeed, the available studies relied on small samples and had high risks of bias (design bias, evaluation bias), and the baseline characteristics of the participants were very heterogeneous. Three 30-year-old studies focusing on foster care in the United States were included [13-15]. Two of them are of interest [13,15]. Braun and Rose [13] compared 269 older participants: 100 lived in foster care, 101 in nursing homes, and 68 at home. After 3 months, Activities of Daily Living (ADL) scores only improved in the foster care group, and costs were lower than those for the other 2 groups (US $28 per day vs US $74 for nursing homes and US $45 for home). There was no difference in morbidity, defined as the number of infections, injuries, skin problems, and hospitalizations. In their randomized study on 112 participants in foster care or nursing homes, followed for 12 months, Oktay and Volland [15] found no difference in mortality rates between settings (32.0% vs 28.8%). Stabilization or improvement of ADL and cognitive status, as well as overall lower health care expenses (25% less), was in favor of foster care. Nevertheless, nursing home residents’ level of satisfaction compared favorably with that of foster family residents. In any case, available data are scarce, assessed with short follow-ups, lack essential clinical outcomes, and definitely need to be updated.

To date, we do not know whether foster care is an alternative to nursing home care, as suggested by the limited existing literature, or a transitional care setting between home care and nursing home care. Epidemiological and medicoeconomic data on older people in foster families are essential to assess the relevance of this type of care, in particular, according to the severity of dementia or the degree of dependence in ADLs. Several issues need to be clarified: what is the residents’ annual hospitalization rate and the associated costs? What is the health care pathway of residents in terms of consultations with general practitioners, specialists, and paramedics? What is the prevalence of geriatric syndromes in this population, and the incidence of mortality? What is the incidence of pneumonia, the leading cause of death and emergency hospitalization in older people? What is the quality of life of the residents and their foster caregivers? The Karukera Study of Ageing in Foster Families (KASAF) is a prospective cohort study, designed to investigate these questions. This longitudinal study will assess the health status and medical and economic data of 250 foster family residents and their foster caregivers over a year.

## Methods

### Overview

The KASAF is a prospective, longitudinal cohort carried out in Guadeloupe (French West Indies), France, among foster families for older adults. This study will collect the socio-medico-economic data of older residents of foster families over a year, with a focus on hospital admissions. We will collect data using face-to-face interviews with the participants and their foster caregivers at baseline and after 6 and 12 months, and using phone interviews with the foster caregivers after 3 and 9 months.

### Objectives

This study is an epidemiological study that aims to explore the characteristics of foster family residents and caregivers. We have identified a number of outcomes that may provide insight on their profile. As hospitalization is the main expensive item in the health trajectories of older people [16], the primary objective of the KASAF is to study hospital admission rates of residents of foster families over a year.

Several secondary objectives are also targeted: length of hospital stays during the 12-month follow-up period; number of consultations with a general practitioner, a medical specialist, or a paramedical professional; global cost of hospital stays; prevalence of geriatric syndromes (frailty, undernutrition, depressive symptoms, incontinence and dependency, sensory impairments; falls; polypharmacy; bedsores; and cognitive impairment); incidence of lower respiratory tract infection; quality of life of foster care recipients and their foster caregivers; and burnout in foster caregivers.

### Study Sample

A total of 250 residents will be recruited over 6 months among the 300 older residents currently in foster families. The primary end point will be the hospitalization rate at 12 months. A 30% hospital admission rate over a year has been reported in the Incidence of pNeumonia and related ConseqUences in nursing home Resident study in nursing home residents [16]. A change in the hospitalization rate from 30% to 15% would be considered as significant from a clinical and public health point of view. For an risk of 5% and 250 participants, the expected CI will be 4.4% for a hospitalization rate of 15% and 5.7% for a 30% rate.

### Study Recruitment

#### Inclusion Criteria

To be eligible for the study, residents must be older than 60 years. They must live in a foster family in Guadeloupe and benefit from the French social security.

#### Exclusion Criteria

The only exclusion criterion is refusal from the resident or his or her legal guardian to participate in the study.

#### Preinclusion Visit

Before the start of inclusions, residents, their families, and the foster caregivers will be informed of the goals and methods of the KASAF via information meetings and emailing campaigns.

The on-site investigator will screen for eligible participants among the residents of the foster family he or she is responsible for and will visit every resident meeting the criteria. He or she will inform them of the aims of the study, the computerized processing of their personal data, the access to their data in the local PMSI (Program for Medicalization of Information Systems [Programme de médicalisation des systèmes d'information]) server, and their right to object to their participation at any given time. An information leaflet will be given to every participant and to the referring professional caregiver identified by the investigator to complete the interviews. Foster caregivers and residents have a right to oppose the collection of their personal data.

#### Data Collection

At inclusion, nurses, geriatricians, and psychologists will collect sociodemographic, health-related, and psychosocial information. The inclusion visit is the first on-site interview and will be followed by 4 visits: 2 on-site visits 6 and 12 months after the inclusion visit, and 2 phone interviews 3 and 9 months after the inclusion visit (Table 1).

The interviewers will be conducted by health care professionals: psychologists, interns, and nurses, trained by the INSERM Team SEPIA (UMR 1219) from the University of Bordeaux, a team having expertise in implementing cohort studies.

**Table 1:** Expected timeline for the KASAF^a^.

Time line	Goal
July-September 2020	Ethics Committee approval Registration on ClinicalTrials.gov
September 2020	Training sessions for the interviewers
November 2020-May 2021	Inclusion period
February 2021-February 2022	3-, 6-, and 9-month follow-up
February 2022-May 2022	End of 12-month follow-up (end of data collection)
June 2022-March 2023	Data analysis
From May 2023	Scientific communications

^a^KASAF: Karukera Study of Ageing in Foster Families.

#### On-site Interviews

Participants will be interviewed at baseline, after 6 months, and after 12 months. At each visit, different function domains will be assessed to measure (1) the ability to perform ADLs (eg, personal care, eating, and mobility) and (2) the ability to perform instrumental ADLs (eg, using the telephone, shopping, and managing finances). In fact, the interview implies both a physical evaluation (grip strength, gait speed, and blood pressure) and a cognitive and a psychological assessment. The estimated time for completion is 30 minutes but may vary depending on the participant’s physical and cognitive status. Each of the participants will remain in the study for further follow-up interviews even if he or she is unable to answer, and reasons for incomplete data will be documented by the interviewers. Interviewers may split the visit into 2 parts to respect the participant’s rhythm and to avoid missing data.

During on-site visits, foster caregivers will provide their own sociodemographic data (as well as information on the caregiving burden borne) and those of the elders to be cared for. Thus, both the quality of life of foster caregivers and the main sociodemographic or health data of the participants (eg, medical history, medication, nutrition, and ADL/Instrumental Activities of Daily Living [IADL] performance) are the primary focus of the study.

#### Phone Interviews

Interviewers will phone the foster caregivers twice to collect data on participants 3 and 9 months after the first visit. This phone call will provide an update on the mortality status of foster elders and health care services (ie, types and frequency of use). In the event of the death of a participant, date and cause of death will be sought and documented whenever possible.

### Outcomes

Information on health care pathway comprises hospitalizations and consultations with general practitioners, specialists, and paramedics.

#### Primary Outcome

The main outcome is the number of hospitalizations over a year. The French hospital discharge database (PMSI) will provide information on hospital admissions. This information will be retrieved with the approval of the participants or their legal representative. Information on hospital admissions will also be collected from the foster caregiver for confirmation.

#### Secondary Outcomes

The secondary outcomes are as follows:

1. Length of hospital stays during the 12-month follow-up period, in terms of days and nights, will be collected. The number of consultations with a general practitioner, a medical specialist, or paramedical professional will be reported on the case report forms (CRFs). This information will mainly be collected during interviews with the foster caregiver using the RUD-Lite scale (Resource Utilization in Dementia questionnaire) [17]. This scale assesses the use of health care resources by people with dementia. The foster caregiver will complete this questionnaire at baseline and at subsequent follow-up visits (T3, T6, T9, and T12).
2. Global cost of hospitalization will be retrieved from the local PMSI databases.
3. Vital status (alive or deceased) will be collected at T3, T6, T9, and T12.
4. For the diagnosis of lower respiratory tract infections, at least two of the following symptoms must be present and a doctor must confirm the clinical evidence: (1) cough (starting or worsening), which may produce phlegm; (2) specific clinical signs during auscultation; (3) fever (≥38 °C); (4) chest pain; (5) rapid, shallow breathing (≥25 per minute); and (6) confusion or increased dependency [16].
5. Geriatric syndromes [18] in participants will be assessed at T0, T6, and T12 as follows:
  Frailty will be assessed using Fried’s phenotype [19], that is, 5 predefined physical frailty criteria such as weight loss, slowness, low physical activity, weakness, and exhaustion. This will be analyzed as a dichotomous variable, that is, 1=frail and 0=not frail.
  The level of cognitive deterioration of the participants will be assessed using the Mini-Mental State Examination (MMSE) [20,21]. This 30-item scale assesses the severity of cognitive deterioration through items such as orientation, learning, attention, arithmetic, memory, language, and constructive praxis. The scale is rated from 0 to 30, reflecting the level of cognitive impairment.
  Depression will be assessed using the Center for Epidemiologic Studies—Depression Scale (CES-D) [22] validated in French by Fuhrer and Rouillon [23]. This 20-item questionnaire assesses depressive symptomatology, including 4 positive polarity items (happiness, self-worth, confidence, and enjoyment). The frequency of symptom occurrence during the past week is assessed by the participant using a 4-point Likert scale (never, occasionally, quite often, and frequently). The score ranges from 0 to 60. The higher the score, the more significant the symptomatology. High depressive symptomatology corresponds to a score above 16. Data will be collected at baseline, T6, and T12. For statistical analyses, the variable will be dichotomized (1=presence of depression and 0=absence of depression).
  The total number of medications taken by participants will be collected directly from the RUD-Lite questionnaire, at T0, T3, T6, T9, and T12, to assess polypharmacy.
  The total number of falls since the last visit will be collected at each follow-up from the foster caregivers.
  The Short Physical Performance Battery [24,25] will help to assess sarcopenia. Three subtests measure lower-extremity functions: keeping balance (feet side-by-side, semitandem, tandem balance position, to be kept for 10 s each); walking 4 m at usual pace, yielding a gait speed ratio (meter per second); standing up and sitting down 5 times as quickly as possible with the arms folded. Each movement is scored 0-4 points out of a total score from 0 to 12, with higher scores showing better physical performance. If the total score is below 8, there is a risk of sarcopenia. For the analyses, the variable will be dichotomized accordingly (1=sarcopenia; 0=no sarcopenia).
  Undernutrition will be assessed with the short version of the Mini Nutritional Assessment [26]. This questionnaire assesses nutritional status. A score below 12 indicates probable undernutrition. For the analyses, the variable will be dichotomized accordingly (1=undernutrition; 0=no undernutrition).
  Sensory impairments (audition and vision) will be collected by directly asking the foster caregiver. Variables will be dichotomized accordingly (1=hearing/vision impairment; 0=hearing/vision impairment).
  The level of independence in ADL and incontinence will be assessed using the Katz scale [27], a 6-item scale assessing independence in 6 basic ADLs: bathing, toileting, transferring, eating, dressing, and incontinence. For each activity, a score of 1 indicates complete autonomy, a score of 0.5 indicates partial autonomy, and a score of 0 indicates complete dependency.
  The level of independence in IADL will be assessed using the Lawton IADL scale [28]. The Lawton IADL scale measures 8 items: using the telephone, shopping, preparing food, housekeeping, doing laundry, using transportation, handling medications, and handling finances. The score is from 0 to 8; a score of 8 indicates total autonomy, and 0 indicates total dependency. In France, the Autonomy, Gerontology Iso-Resources (AGGIR) questionnaire has been developed by public authorities to quickly assess the degree of dependency of elderly people and assign a dependency group, ranging from 0 (bedridden, completely dependent) to 6 (no help required, the person is autonomous in everyday activities).
  Psychological and behavioral symptoms in dementia will be assessed with the short version of the NeuroPsychiatric Inventory or NPI [29,30]. This scale assesses the psychological, behavioral manifestations (agitation, hallucinations, apathy, etc), neurovegetative symptoms (sleep and appetite), and the impact on the professional’s workload.
6. Feeling of loneliness will be captured with the 14th item of the CES-D [22,23]: I felt lonely. The answer possibilities are never, occasionally, often, and frequently. The answer “often” or “frequently” indicates a feeling of loneliness.
7. Risk of developing pressure ulcers will be assessed with the Braden scale, and pressure ulcers will be classified according to the National Pressure Ulcer Advisory Panel, European Pressure Ulcer Advisory Panel, and Pan Pacific Pressure Injury Alliance [31].
8. Quality of life and adaptation will be assessed as follows:
  - Quality of life of the participant will be assessed at baseline, T6, and T12 using the Questionnaire Quality of Life—Alzheimer’s Disease (QoL-AD) [32,33]. This 13-item questionnaire assesses the participant’s physical condition, mood, relationships with friends and family, financial difficulties, and overall quality of life. The questionnaire is to be administered to both seniors and caregivers, and thus is based on both self-rated health problems and proxy ratings from foster families. The items are evaluated using a 4-point ordinal scale ranging from poor to excellent, with a total score ranging from 13 to 52. For the weighted score, the participant’s score is multiplied by 2, the caregiver’s score is added, and the total is divided by 3 to bring the score back to the initial scale. In the proxy version, the foster caregiver will be asked to try to give the answer they think the participant would give.
  - Health-related quality of life of the participant will be assessed with the EQ-5D questionnaire [34]. This questionnaire assesses health conditions. It is the most widely used questionnaire in medicoeconomic analyses. It allows health outcomes to be assessed in terms of quality-adjusted life-years (QALYs). This indicator summarizes the length and quality of life in a single figure. The result of a cost-utility analysis is expressed in terms of the cost of gaining one QALY (ie, 1 year lived in perfect health). To estimate QALY, health-related quality of life is designed on a continuum from 0 (death) to 1 (perfect health). Each health status is thus assigned a weight of 0 to 1; the higher the score, the higher the corresponding quality of life.
    - On the first page, the participant has to indicate, for each of the 5 dimensions assessed (mobility, independence, routine activities, pain or discomfort, and anxiety or depression), the state in which he or she finds himself or herself, by choosing 1 of the 3 options with increasing severity: no problems, problems, and extreme problems. The answers can be combined to describe the residents’ health status, using a 5-digit number corresponding to the 5 answers.
    - On the second page, the participant assesses his or her overall health using a standard vertical 20 cm visual analog scale (EuroQol visual analog scale), graduated from 0 to 100, from “best imaginable state of health” to “worst imaginable state of health.”
    - The score will be collected at T0, T6, and T12. The interviewer will use both the participant version and the proxy version. In the proxy version, the foster caregiver will be asked to try to give the answer they think the participant would give.
  - Adaptation to the nursing home setting will be assessed at baseline, T6, and T12 using the Castonguay and Ferron scale [35]. This is a 17-item scale designed to identify who do not experience a favorable adaptation to the living conditions in nursing homes. The participants answer yes or no to a series of statements about their daily life and habits in the residence. A score of 16 or 17 indicates a good general adaptation to life in the residence, whereas a score of 11 or less indicates significant difficulties.
  - Foster caregivers’ quality of life will be assessed with the Medical Outcomes Study Short-Form General Health Survey—36 items (SF-36) [36], a widely validated health-related quality of life questionnaire that uses questions on physical, social, and psychological domains.
  - Symptoms of burnout will be assessed with the Professional Quality of Life (Pro-QoL) scale [37]. This 30-item questionnaire yields 3 domain-specific scores: compassion fatigue (ie, the pleasure of being able to do your job well), work satisfaction, and burnout in helping professionals.

Table 2 shows an overview of data collection at each follow-up.

**Table 2:** Data collection over time in the KASAF^a^.

			Baseline (T0)		Phone interview (T3 months)		Follow-up (T6 months)		Phone interview (T9 months)		Final visit (T12 months)
**Older residents—self-rated questionnaires and anthropometric measures**											
	CES-D scale^b^	X^c^				X				X	
	QoL-AD scale^d^	X				X				X	
	EQ-5D-3L scale^e^	X				X				X	
	Adaptation to the institution scale	X				X				X	
	MMSE score^f^	X				X				X	
	SPPB scale^g^	X				X				X	
	CDR score^h^	X				X				X	
**Foster caregivers—proxy-rated questionnaires about the resident**											
	Medical characteristics	X				X				X	
	Prescribed drugs	X				X				X	
	NPUAP-EPUAP^i^ score^i^	X				X				X	
	Braden scale	X				X				X	
	ADL^j^ and IADL^k^ scales	X				X				X	
	AGGIR score^l^	X				X				X	
	MNA-SF scale^m^	X				X				X	
	NPI-ES scale^n^	X				X				X	
	QoL-AD scale	X				X				X	
	EQ-5D-3L score	X				X				X	
	RUD-Lite scale^o^	X		X		X		X		X	
	Health events			X		X		X		X	
	Alive/dead status			X		X		X		X	
**Foster caregivers—self-rated questionnaires**											
	Sociodemographic characteristics	X				X				X	
	Pro-QoL scale^p^	X				X				X	
	SF-36 scale^q^	X				X				X	

^a^KASAF: Karukera Study of Ageing in Foster Families.

^b^CES-D: Center for Epidemiologic Studies—Depression Scale.

^c^X: information that will be collected during the indicative period.

^d^QoL-AD: Questionnaire Quality of Life—Alzheimer’s Disease.

^e^EQ-5D-3L: EQ-5D three-level version.

^f^MMSE: Mini-Mental State Examination.

^g^SPPB: Short Physical Performance Battery.

^h^CDR: Clinical Dementia Rating.

^i^NPUAP-EPUAP: National Pressure Ulcer Advisory Panel–European Pressure Ulcer Advisory Panel.

^j^ADL: Activities of Daily Living.

^k^IADL: Instrumental Activities of Daily Living.

^l^AGGIR: Autonomy, Gerontology Group Iso-Ressources.

^m^MNA-SF: Mini Nutritional Assessment Short-Form.

^n^NPI-ES, NeuroPsychiatric Inventory for Health Staff.

^o^RUD-Lite: Resource Utilization in Dementia—Lite Version.

^p^Pro-QoL: Professional Quality of Life.

^q^SF-36: Short-Form General Health Survey—36 items.

#### Expected Timeline

The first participant was included on November 16, 2020. The 1-year follow-up was ended in June 2022 (Table 1).

### Patient and Public Involvement

There was no patient or public involvement in the design and conduct of this study. Nevertheless, we have had discussions with associations of foster caregivers who would like to use our results to strengthen their professional training.

### Data Analysis

First, the analysis will describe the number of included participants, the inclusion curve (evolution of the number of people included between the first and last inclusion), the number of theoretical visits, the number of visits actually made, and the ratio of both (number of visits made or number of theoretical visits).

A description of the rate and causes of death and study dropout will also be provided.

The following quantitative variables described in terms of number of staff, mean, and interindividual SD and 95% CI of the mean, median, range, and IQR values: number of hospitalizations, total length of hospitalization, total cost related to hospitalizations, total number of consultations with a general practitioner, total cost of low respiratory infections, total number of falls, MMSE score, total number of drugs, NPI score, QoL-AD score, SF-36 score, and Pro-QoL score.

Qualitative variables (ordinal: AGGIR grid, feeling of loneliness; dichotomous: vital status; presence or absence: frailty syndrome, depression, undernutrition, visual impairment, hearing impairment, incontinence, ADL dependency, IADL dependence) of participants at inclusion will be described in terms of frequency and number.

Comparisons between the 3 times (T0, T6, and T12) will be performed using repeated-measures ANOVA for quantitative variables and the Cochran-Armitage test for trend for qualitative variables.

Statistical analyses will be performed using the RStudio software (version 2.7; Posit Software).

### Quality Control

During the study, quality control will be carried out by the project manager and the clinical research assistant. All interviewers will be required to attend training on data collection and data reporting. Under no circumstances should the names or addresses of the individuals concerned be made clear.

The electronic CRF will allow single data entry, which will be double checked by the investigators. Data management will be performed using the Capture System software (Clinsight). The Clinsight web-based module will allow remote access to the database, with a 128-bit SSL encryption. Each interviewer will have a personal and unique name and access code that will allow him or her to enter and correct data—everything is documented and saved every day on another server.

### Ethics and Dissemination

The KASAF is an observational study involving human participants without any identified risk for the participants’ safety. In accordance with this status, the need for a signed consent form has been waived off by the regulating bodies (law 2012-300). Residents will be provided with an information leaflet outlining the key points of the study. Participation is voluntary and residents can refuse to participate or discontinue participation at any time without prejudice. Considering the high proportion of family foster residents with cognitive impairment, the on-site investigator will ensure that the residents have understood the implications of their participation. If the resident is under guardianship and/or is unable to understand, nonopposition from the legal guardian or a documented contact person will be sought as well.

Nonopposition or opposition will be documented in the participant’s medical file.

This study will be carried out in accordance with state laws regulating research involving human participants and with the Helsinki declaration.

The KASAF was registered under the RGB-ID number 2020-A00620-39 and was approved by the French Sud Méditerranée III Ethics Committee on July 1, 2020. Data analysis will be performed on-site (University Hospital, Guadeloupe). All documents related to this research will be archived for 15 years after completion of the study.

## Results

Recruitment opened in September 2020 and ended in May 2021, with 109 older people recruited and 56 respective foster caregivers. The 1-year follow-up was ended in June 2022. Data analyses are ongoing, and the first results are expected to be published in May 2023.

## Discussion

Foster families are a potentially innovative way to accommodate older people with dependency. Epidemiological data from the study will describe the clinical characteristics of older people in foster families. Beyond mortality and morbidity, the numerous outcomes will allow assessing the evolution of geriatric syndromes over time. In addition, this study will also look at the sociodemographic profile of foster family caregivers—who are not necessarily health professionals—in terms of age, life course, quality of life, or burnout. It may offer a basis for specific training courses and help the authorities to better characterize the requirements an applicant must meet to become a fully-fledged foster caregiver.

This descriptive study could help define the place of the foster family in the transition from frail elderly to dependent elderly status. Indeed, foster family could also be an intermediate stage between home care and nursing home care. The epidemiological data obtained will be compared with data from other management systems. In this perspective, we will conduct a twin study in nursing home, the KArukera Study of Aging in EHPAD (KASEHPAD) study, which will take place in 14 nursing homes in Guadeloupe. Both studies will have similar designs and objectives. The protocol for the KASEHPAD study has been registered on ClinicalTrials.gov (NCT04587466). If patient characteristics are similar, it will be possible to compare mortality, hospitalization rates, and all variables of interest, including cost of care, between these 2 care settings. Depending on the profile of the KASAF’s participants, we could also compare foster care with home care or residential care facilities [38]. The collection of epidemiological data in foster family is therefore an essential step to carry out randomized studies between these different care pathways, with the aim of determining the most effective and efficient care.

Foster families have never been formally assessed, the most robust results dating back to 30 years. In theory, it is less expensive than nursing homes for the community, but in the absence of scientific studies on the associated health outcomes (number of hospitalizations, deaths, etc), this model has not been validated and remains rare in metropolitan France. Yet, foster caregivers could be valuable assets in health care, thanks to their intimate knowledge of older persons and their pathologies, compared with nursing home staff, which have a high turnover, thus reducing avoidable hospitalizations and medical consultations.

Moreover, in the event of a health crisis such as the one experienced with the COVID-19 pandemic, measures of isolation and social distancing are much easier to implement, as foster family residents live in small family units, usually have single rooms, with less comings and goings than in nursing homes. The collected data will be used for exploring the characteristics of the foster home residents with aims that might not be considered today (1) because they are out of topic and (2) because science evolves.

This study presents limitations. We will not have access to data from informal caregivers, who could provide different insights on the participant’s state of mind or daily life. Also, cost analyses will focus only on hospitalization costs. This is why an ancillary study will be carried out after the end of the KASAF, using information from the national health insurance database. This study will accurately assess the cost of the entire health care pathway, including nursing, medical transportation, and other health care expenses. Finally, the KASAF does not include randomized interventions versus nursing home limiting causal interpretations of the results.

We hope that our results will help introduce more rationality into social and economic debates, sometimes led by accounts of personal experiences and extrapolated indicators, often for lack of robust data, and help ensure that public health policies are based on sound scientific evidence. Indeed, our data will be used to support the coordination of institutional funding bodies, especially resource allocation.

## Abbreviations

ADL: Activities of Daily Living
AGGIR: Autonomy, Gerontology Group Iso-Ressources
CES-D: Center for Epidemiologic Studies—Depression Scale
CRF: case report form
IADL: Instrumental Activities of Daily Living
KASAF: KArukera Study of Ageing in Foster Families
KASEHPAD: KArukera Study of Aging in EHPAD
MMSE: Mini-Mental State Examination
NPI: NeuroPsychiatric Inventory
PMSI: Program for Medicalization of Information Systems
Pro-QoL: Professional Quality of Life
QALY: quality-adjusted life-year
QoL-AD: Questionnaire Quality of Life—Alzheimer’s Disease
RUD-Lite: RUD-Lite: Resource Utilization in Dementia—Lite Version
SF-36: Short-Form General Health Survey—36 items

## Data Availability

The data sets generated during and analyzed in this study are available from the corresponding author on reasonable request.
